# FGFR2 alteration as a potential therapeutic target in poorly cohesive gastric carcinoma

**DOI:** 10.1186/s12967-021-03079-8

**Published:** 2021-09-22

**Authors:** Yue Wang, Tao Shi, Xuan Wang, Jinwei Hu, Lixia Yu, Qin Liu, Nandie Wu, Baorui Liu, Jia Wei

**Affiliations:** 1 The Comprehensive Cancer Centre of Nanjing Drum Tower Hospital, The Affiliated Hospital of Nanjing University Medical School & Clinical Cancer Institute of Nanjing University, No. 321, Zhongshan Road, Nanjing, 210008 China; 2 The Comprehensive Cancer Centre of Nanjing Drum Tower Hospital, Clinical College of Nanjing Medical University, No. 321, Zhongshan Road, Nanjing, 210008 China; 3 OrigiMed, No. 115, Xinjun Ring Road, Shanghai, 201114 China

**Keywords:** Gastric cancer, Poorly cohesive, Next-generation sequencing, Gene rearrangement, Fibroblast growth factor receptors 2

## Abstract

**Background:**

Poorly cohesive (PC) is a unique histologic subtype of gastric cancer (GC), with an increasing incidence in recent years. However, the molecular characteristics and therapeutic targets of PC GC are not yet well studied and there are no effective therapies for these patients.

**Methods:**

Formalin fixed paraffin embedded (FFPE) samples of 556 GC patients, including 64 PC GC, were collected for next-generation sequencing (NGS). Clinical characteristics and genomic profiling were analyzed. FGFR2 expression was detected by quantitative real time polymerase chain reaction (qRT-PCR) and immunohistochemistry (IHC). FGFR2 inhibitors response was studied in vitro.

**Results:**

Among 556 GC patients, PC GC patients were younger (P = 0.004), had lower tumor mutation burden (TMB-L) (P = 0.001) than non-PC GC. The top 10 most frequently mutated genes in PC GC were TP53 (48%), CDH1 (31%), ARID1A (14%), FGFR2 (14%), ERBB2 (9%), CDKN2A (9%), FGF3 (8%), LRP1B (9%), FGF19 (8%) and FGF4 (8%). Noticeably, FGFR2 is more frequently mutated than non-PC GC (14% vs. 6%, P = 0.037), including copy number variants (CNVs, 12.5%) and gene rearrangements (3.1%, FGFR2/VTI1A and FGFR2/TACC2). Former studies have confirmed that gain of copy number could increase FGFR2 expression and sensitivity to FGFR2 inhibitors in GC. However, no research has verified the function of FGFR2 rearrangements in GC. Our results showed that cell lines of GC transfected with TACC2-FGFR2 fusion had increased mRNA and protein expression of FGFR2, and were more sensitive to FGFR2 inhibitors. FGFR2 inhibitors might be a new therapeutic target for PC GC. In addition, we found patients of PC GC harboring gene rearrangements (n = 9) had poorer overall survival (OS) in comparison with patients without any gene rearrangement (n = 19) (16.0 months vs 21.0 months, P = 0.043). Gene rearrangement might be an adverse prognostic factor for PC GC patients.

**Conclusions:**

FGFR2 alterations were recurrent in PC GC and FGFR2 inhibitors might be a new therapeutic target for PC GC.

**Supplementary Information:**

The online version contains supplementary material available at 10.1186/s12967-021-03079-8.

## Background

Gastric cancer (GC) is the fifth most common cancer and the third leading cause of cancer mortality in the word [1]. Numerous studies have shown that GC is histologically and genetically heterogeneous. The WHO classified GC into papillary adenocarcinoma, tubular adenocarcinoma, mucinous adenocarcinoma, poorly cohesive (PC) carcinoma, mixed adenocarcinoma and other rare subtypes [2]. In recent years, a decrease occurred in the overall incidence of GC, however, the proportion of GC with WHO PC histology is increasing [3]. PC GC is defined as tumor composed of isolated or small groups of tumor cells by the WHO classification [3]. Compared to other GC subtypes, PC GC is poorly differentiated and easy to metastasized. None of current treatments, including chemotherapy, radiotherapy, targeted therapy and immunotherapy, has shown good results [4, 5].

Although the histopathological classifications currently remain the most commonly used for therapy decision making in the clinical setting, molecular classifications have been developed to guide future treatment development [5]. Several different molecular GC classification systems have been proposed these years, attempting to relate molecular features to histological phenotypes and clinical features [6, 7]. In 2014, The Cancer Genome Atlas (TCGA) research network proposed four molecularly distinct GC subtypes: Epstein–Barr virus infected (EBV), microsatellite instability (MSI), genomically stable (GS), and chromosomal instability (CIN) [8]. Also, in 2015, the Asian Cancer Research Group classified GC into four molecular subtypes: mesenchymal-like, microsatellite-unstable, the tumor protein 53 (TP53)-active and TP53-inactive types [9]. However, there are few detailed studies on the mutational spectrum of PC GC. Further investigation of the genetic alterations may provide useful information to explore new therapies for PC GC patients.

In this study, we compared the clinical characteristics and genomic profiling of PC GC patients and non-PC GC patients. Furthermore, we investigated whether genetic alterations are associated with patient prognosis in PC GC. Finally, we tried to seek new therapies according to specific genetic alterations of PC GC.

## Methods

### Patients and samples

We collected 556 patient samples of gastric adenocarcinoma. All histopathologic diagnoses were reviewed by at least two senior pathologists independently. Clinical information was retrospectively collected and the overall survival (OS) was measured from the date of surgery to the date of death or the last follow-up visit. The last follow-up date was August 8th, 2016. This study was conducted in accordance with the code of ethics of the World Medical Association (Declaration of Helsinki) and approved by the Ethics Committee of Nanjing Drum Tower Hospital (No. 2016-196-01).

### Nucleic acid preparations and next-generation sequencing

Formalin fixed paraffin-embedded (FFPE) tissues obtained from patients with GC were collected for NGS in a 450-gene panel assay, whose accuracy has been verified before (Additional file 1: Figure S1). DNA was extracted from the unstained FFPE sections with tumor content of no less than 20% and was fragmented to ~ 250 bp by sonication. A library was constructed using the KAPA Hyper Prep Kit (KAPA Biosystems) and hybridization capture was performed with a custom panel containing individually synthesized 5ʹ-biotinylated DNA probes. The capture probes target exons of 450 cancer-related genes and frequently rearranged introns of 38 genes. Paired-end sequencing was performed according to the manufacturer’s protocols. Genomic alterations, including single base substitutions, short and long insertions/deletions (INDELs), copy number variations, and gene rearrangements, were assessed using the OrigiMed-pipeline. Genomic alterations relevant for cancer immunotherapy, which included TMB levels and MSI, were also evaluated [10, 11]. Mutation signature was predicted using a public software deconstuctSigs. All substitution mutations were classified into 96 kinds of trinucleotides and the frequency of each was precisely calculated as the characteristic signature of these samples. The signature was then compared with the typical 30 signatures from COSMIC to identify the most similar combination and the percentage of each contributed [12].

### Cell lines

To verify the function of TACC2-FGFR2 fusion, we generated gastric cancer cell lines with stable expression of TACC2-FGFR2. The Lenti-EF1a-EGFP-pGK-Puro retroviral vectors containing the particular fusion genes were transfected into 293 T cells to produce virus. MKN45 and NUGC4 cells were then infected with the viral supernatant containing expression constructs. Stable transfectants were obtained and maintained under selection pressure by puromycin dihydrochloride (2 µg/ml, Beyotime, ST551-10 mg). Under selection conditions, clones were picked and maintained.

### Quantitative real time polymerase chain reaction (qRT-PCR) analysis

Total RNAs were extracted by TRIzol™ Reagent (Thermo Fisher Scientific, 15596018), then dissolved in RNase-free water. cDNA synthesis is performed in the first step using total RNA, Random Primers (Thermo Fisher Scientific, 48190011) and dNTP Mix (10 mM ea, 18427013), 5 min at 65 °C. In the second step, PCR is performed in a separate tube using probes specific for the gene of interest, M-MLV Reverse Transcription (200 U/ul, 28025013) and RNase OUT™ Recombinant Ribonuclease Inhibitor (Thermo Fisher Scientific, 10777019), following the procedures of 10 min at 25 °C, 45 min at 37 °C, 10 min at 70 °C, and then 4 °C. TaqMan™ Fast Advanced Master Mix (Thermo Fisher Scientific, 444556) was used with the following PCR parameters, 1 cycle of 20 s at 95 °C, 40 cycles of 1 s at 95 °C and 20 s at 60 °C using QuantStudio™ 7 Flex (Applied Biosystems). Probes used in this study are FGFR2 (Thermo Fisher Scientific, Hs01552918_m1) and β-actin (Thermo Fisher Scientific, Hs99999903_m1).

### Immunohistochemical staining of tumor tissues

The expression of PD-1, PD-L1, CD3, FGFR2 in tumors was evaluated via immunohistochemical analysis (anti-PD-1, 1:100, NAT105, Cell Marque; anti-PD-L1, 1:100, SP142, Spring Bio; anti-CD3, 1:500, CD3-565-L-CE, Leica/Novocastra; anti-FGFR2, 1:200, ab58201, Abcam). The PD-1, PD-L1 is observable in the cytoplasm or on the membrane of the tumor cell or the TILs. The immunoreactivity of PD-1, PD-L1 was evaluated semiquantitatively according to the percentage and intensity of positive cells. Specimens in which PD-1 or PD-L1 were observed in more than 1% of tumor cells or immune cells were considered PD-1 or PD-L1 positive. CD3 was detected in the nuclei of the TILs. The distribution of CD3+ TILs was observed in the areas with the highest density of TILs first at low magnification. The amount of positive TILs was then recorded at high magnification (HPF 400× magnification). The number of CD3+ TILs was determined in 30 random high power fields in each section.

### Fluorescence in situ hybridization (FISH)

Two-color FISH was performed on 2 µm thick sections from formalin-fixed, paraffin-embedded tumor tissues with FGFR2 rearrangements and paired normal tissues. Before hybridization, sections were deparaffinized, dehydrated in 100% ethanol, and air-dried. Commercially available, locus-specific FGFR2 probe (anbiping, F.01197-01) were used according to the manufacturer’s recommendations. 377-kb Spectrum Green directly labeled fluorescent DNA in the 3ʹ of FGFR2 and 446-kb Spectrum Red directly labeled fluorescent DNA in the 5ʹ of FGFR2.

### Drug sensitivity test

Cell counting kit-8 (CCK-8) (Vazyme, A311-01/02) assay was used to estimate drug response. Briefly, 2500 cells were seeded each well of 96-well plates with 100 μl of 10% FBS 1640 medium, and treated with BGJ398 (Selleck, S2183), AZD4547 (Selleck, S2801) or Erdafitinib (Selleck, S8401) on the 2nd day. After additional days of incubation, 10 μl CCK-8 was added into each well and incubated for 2 h. Afterwards, absorbance was measured at 450 nm with microplate reader. The IC50 values were calculated with nonlinear regression analysis by using GraphPad.

### Statistical analysis

Statistical analysis was performed by SPSS statistics software, version 25.0 (SPSS, Chicago, IL, USA). The continuous variables were tested for normal distribution before analyzing by t test. The categorical variables were taken with the Pearson’s Chi-square test (or Fisher’s exact test). Impact of clinical characteristics and genetic alterations on survival outcomes were estimated by using Kaplan–Meier method, Cox proportional hazard modeling. A two-tailed P value of less than 0.05 was regarded as statistically significant.

## Results

### Clinical characteristics and genomic profiling of PC GC and non-PC GC patients

In this study, a total of 556 GC samples were included. Among all GC patients, 64 (11.5%) are diagnosed as PC GC and 492 (88.5%) are non-PC GC. We compared the clinical characteristics between PC GC and non-PC GC patients (Table 1). It showed that PC GC patients were younger than non-PC GC (proportion of patients younger than 60 years old was 65.6% vs. 46.3%, *P* = 0.004) (Table 1). Also, PC GC patients are more likely to be female, although there was no significant difference (proportion of female patients was 40.6% vs. 32.7%, *P* = 0.208) (Table 1).

**Table 1:** Clinical characteristics and genomic profiling of PC GC and non-PC GC patients (N = 556)

	No. of patients	PC n = 64 (%)	Non-PC n = 492 (%)	*P*^*b*^
Age (years)	556			*0.004*
< 60	270	42 (65.6%)	228 (46.3%)	
≥ 60	286	22 (34.4%)	264 (53.7%)	
Gender	556			0.208
Male	369	38 (59.4%)	331 (67.3%)	
Female	187	26 (40.6%)	161 (32.7%)	
MSI status	545			0.085
MSS	516	64 (100%)	452 (91.8%)	
MSI-H	29	0 (0%)	29 (5.9%)	
TMB^a^	556			*0.001*
TMB-L	411	58 (90.6%)	353 (71.7%)	
TMB-H	145	6 (9.4%)	139 (28.3%)	
Gene SNVs and INDELs				
TP53	349	30 (46.9%)	319 (64.8%)	*0.005*
CDH1	80	18 (28.1%)	62 (12.6%)	*0.001*
ARID1A	98	9 (14%)	89 (18.1%)	0.426
ERBB2	29	6 (9.4%)	23 (4.7%)	0.196
CDKN2A	17	4 (6.3%)	13 (2.6%)	0.234
RHOA	32	4 (6.3%)	28 (5.7%)	1.000
SMAD4	33	2 (3.1%)	31 (6.3%)	0.465
Gene CNVs				
FGFR2	33	8 (12.5%)	25 (5.1%)	*0.037*
CCNE1	63	2 (3.1%)	61 (12.4%)	*0.028*
ERBB2	42	1 (1.6%)	41 (8.3%)	0.094
VEGFA	28	1 (1.6%)	27 (5.5%)	0.295
Gene rearrangements				
FGFR2	8	2 (3.1%)	6 (1.2%)	0.518

*CNV* copy number variations, *INDEL* short and long insertion/deletion, *MSI* microsatellite instability, *MSI-H* microsatellite instability-high, *MSS* microsatellite stable, *PC* poorly cohesive, *SNV* single nucleotide variant, *TMB* tumor mutation burden, *TMB-H* tumor mutational burden-high, *TMB-L* tumor mutational burden-low

^a^Tumors with TMB < 10 Muts/Mb are defined as TMB-L, while ≥ 10 Muts/Mb defined as TMB-H

^b^Pearson’s Chi-square test (or Fisher’s exact test) was used in statistical analyses. Values in italic are statistically significant

**Fig. 1:**
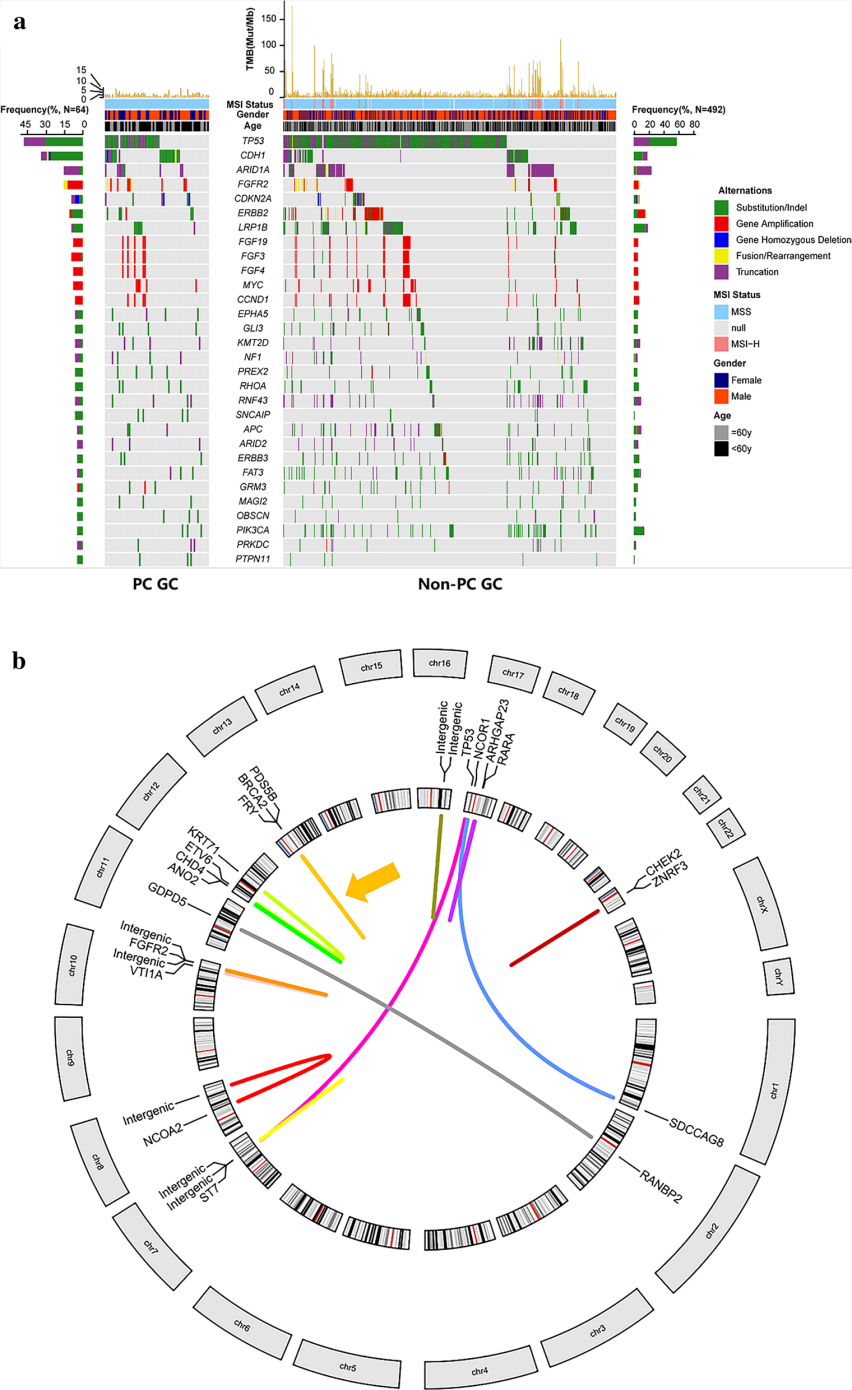
**a** Overview of recurrent somatic genomic alterations in GC patients. The patient samples are shown on the x-axis. Information of mutation rate, MSI status, gender, and patient age are shown on the top of y-axis, followed by the key genetic alterations including significant mutated genes. Frequency of each alteration were illustrated on the left or right of the mutation heat plot. **b** Overview of gene rearrangements in 64 gastric PC GC patients. Each line connecting two genes represented one gene rearrangement, and each color represented one patient. In all, 13 gastric PC GC patients had gene rearrangements. Orange arrow indicated the patient who had two gene rearrangements and they overlapped each other

Formalin fixed paraffin-embedded (FFPE) tissues obtained from these 556 GC patients were collected for NGS in a 450-gene panel assay (Fig. 1a). Microsatellite instability (MSI) status was identified based on mismatch repair (MMR) gene expression, while tumor mutation burden (TMB) was defined as mutations per megabase (Muts/Mb). NGS analysis revealed all 64 gastric PC GC patients were MSS, while 29 (5.9%) non-PC GC patients were MSI-H (*P* = 0.085) (Table 1). Similarly, only 9.4% (6/64) PC GC patients had high TMB (≥ 10 Muts/Mb) while the proportion among non-PC GC patients was 28.3% (139/492) (*P* = 0.001) (Table 1).

The top 10 most frequently mutated genes among 64 gastric PC GC patients were TP53 (48%), CDH1 (31%), ARID1A (14%), FGFR2 (14%), CDKN2A (9%), ERBB2 (9%), LRP1B (9%), FGF19 (8%), FGF3 (8%), and FGF4 (8%), compared to TP53 (66%), ARID1A (20%), LRP1B (16%), CDH1 (14%), PIK3CA (12%), FAT4 (10%), TGFBR2 (10%), KRAS (10%), APC (9%) and KMT2D (8%) in non-PC GC patients (Fig. 1a). Genomic alterations including single nucleotide variants (SNVs), INDELs, copy number variations (CNVs), gene fusions and rearrangements were assessed. We respectively compared the most frequent SNVs and INDELs, CNVs and rearrangements between PC GC and non-PC GC. SNVs and INDELs of TP53 (64.8% vs 46.9%, *P* = 0.005), CNVs of CCNE1 (12.4% vs 3.1%, *P* = 0.028) occurred more frequently in non-PC GC while SNVs and INDELs of CDH1 (12.6% vs 28.1%, *P* = 0.001), CNVs of FGFR2 (5.1% vs 12.5%, *P* = 0.037) occurred more frequently in PC GC (Table 1).

### The value of gene rearrangements in predicting prognosis for PC GC

**Fig. 2:**
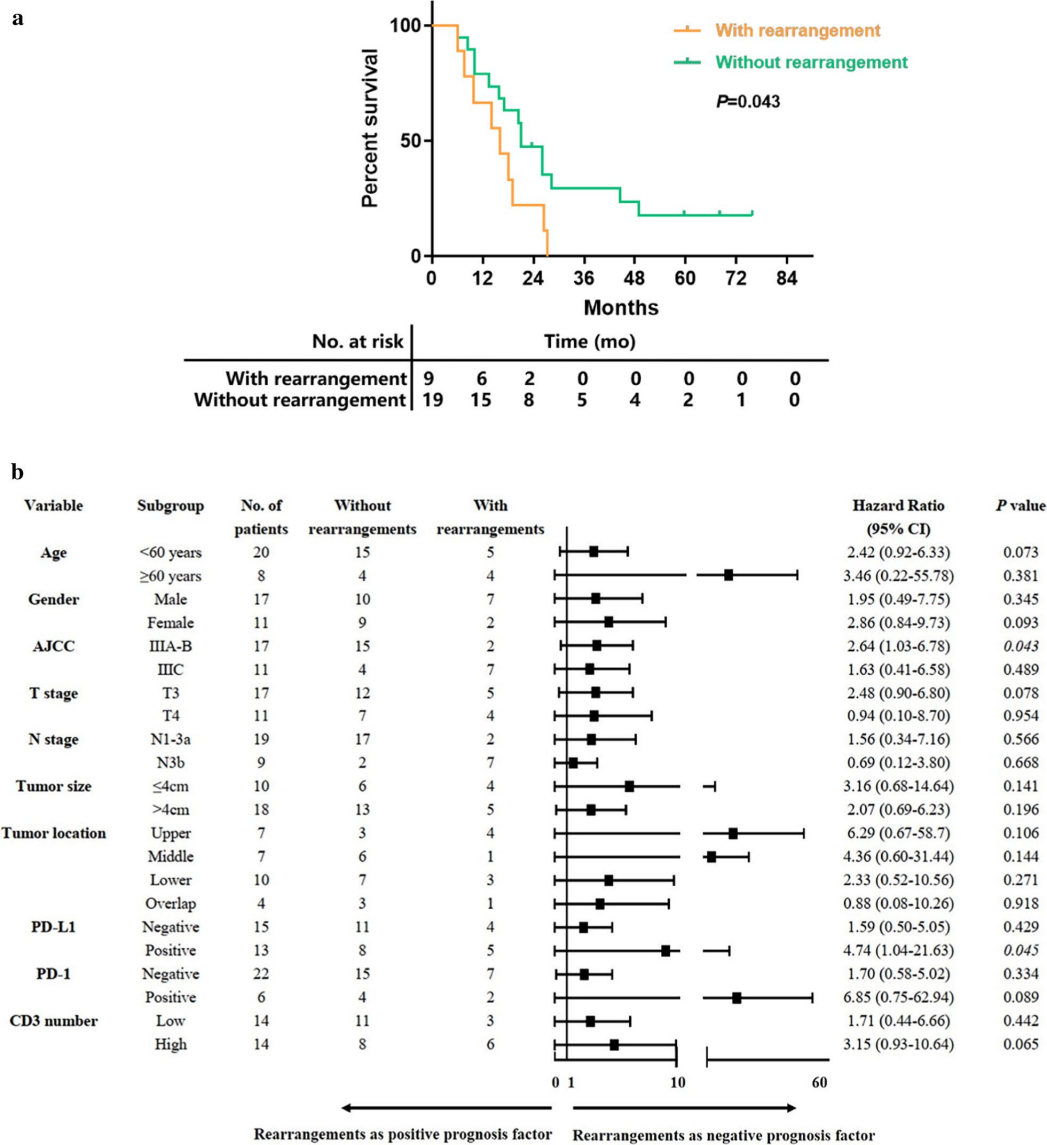
**a** Kaplan–Meier estimates of survival probability. The survival curve of patients with and without rearrangements. **b** Subgroup analysis of PC GC patients with and without rearrangements

We detected gene rearrangements in 20.3% (13/64) PC GC patients (Fig. 1b). Specially, FGFR2 rearrangements (FGFR2/VTI1A and FGFR2/TACC2) were recurrently detected in 3.1% (2/64) PC GC tumor samples. We collected clinical characteristics and prognostic information of 28 PC GC patients from Drum Tower Hospital. It demonstrated that PC GC patients with gene rearrangements had a higher N stage (*P* = 0.001) and a higher tumor stage (*P* = 0.010) defined by 8th American Joint Committee on Cancer (AICC) criterion (Table 2). Moreover, patients harboring gene rearrangements (n = 9) had a shorter overall survival (OS) in comparison with patients without any gene rearrangement (n = 19) (16.0 months vs 21.0 months, *P* = 0.043) (Fig. 2a). To further explore the risk factors related to survival outcome of PC GC, we employed univariate and multivariate Cox regression analyses to identify protective or adverse prognostic factors. As shown in Additional file 1: Table S1, independent prognosis factors of PC GC identified in the univariate Cox regression are age over 60 years old (HR = 2.630, 95% CI 1.104–6.268, *P* = 0.029), higher tumor stage (HR = 2.506, 95% CI 1.026–6.122, *P* = 0.044), higher N stage (HR = 3.789, 95% CI 1.507–9.527, *P* = 0.005) and overlapped tumor location (HR = 4.543, 95% CI 1.089–18.948, *P* = 0.038). Also, patients with gene rearrangements was adverse prognosis factor for PC GC patients, although without significant difference (HR = 2.384, 95% CI 0.993–5.723, *P* = 0.052). Results of multivariate Cox regression suggested that among PC GC patients, age over 60 years old (HR = 3.083, 95% CI 1.114–8.531, *P* = 0.030) were considered as adverse prognosis factor. In addition, Cox’s regression model was separately used to estimate HR and 95% CI in each subgroup (Fig. 2b). Among IIIA-B stage (HR = 2.64, 95% CI 1.03–6.78, *P* = 0.043), PD-L1 positive (HR = 4.74, 95% CI 1.04–21.63, *P* = 0.045) PC GC patients, gene rearrangement was a negative factor for overall survival (Fig. 2b).

**Table 2:** Clinical characteristics of PC GC with and without gene rearrangements

	No. of patients	With rearrangements n = 9 (%)	Without rearrangements n = 19 (%)	*P*^*a*^
Age (years)				0.371
< 60	20	5 (55.6%)	15 (78.9%)	
≥ 60	8	4 (44.4%)	4 (21.1%)	
Gender				0.249
Male	17	7 (77.8%)	10 (52.6%)	
Female	11	2 (22.2%)	9 (47.4%)	
AJCC				*0.010*
IIIA–B	17	2 (22.2%)	15 (78.9%)	
IIIC	11	7 (77.8%)	4 (21.1%)	
T stage				1.000
3	17	5 (55.6%)	12 (63.2%)	
4a–4b	11	4 (44.4%)	7 (36.8%)	
N stage				*0.001*
1–3a	19	2 (22.2%)	17 (89.5%)	
3b	9	7 (77.8%)	2 (10.5%)	
Tumor size (cm)				
≤ 4	10	4 (44.4%)	6 (31.6%)	0.677
> 4	18	5 (55.6%)	13 (68.4%)	
Tumor location				0.370
Upper	7	4 (44.4%)	3 (15.8%)	
Middle	7	1 (11.1%)	6 (31.6%)	
Lower	10	3 (33.3%)	7 (36.8%)	
Overlap	4	1 (11.1%)	3 (15.8%)	
PD-L1				0.689
Negative	15	4 (44.4%)	11 (57.9%)	
Positive	13	5 (55.6%)	8 (42.1%)	
PD-1				1.000
Negative	22	7 (77.8%)	15 (78.9%)	
Positive	6	2 (22.2%)	4 (21.1%)	
CD3				0.420
Low	14	3 (33.3%)	11 (57.9%)	
High	14	6 (66.7%)	8 (42.1%)	

*AJCC* American Joint Committee on Cancer, *PC* poorly cohesive

^a^Fisher’s exact test was used in statistical analyses. Values in italic are statistically significant

### Function of TACC2-FGFR2 fusion in PC GC

**Fig. 3:**
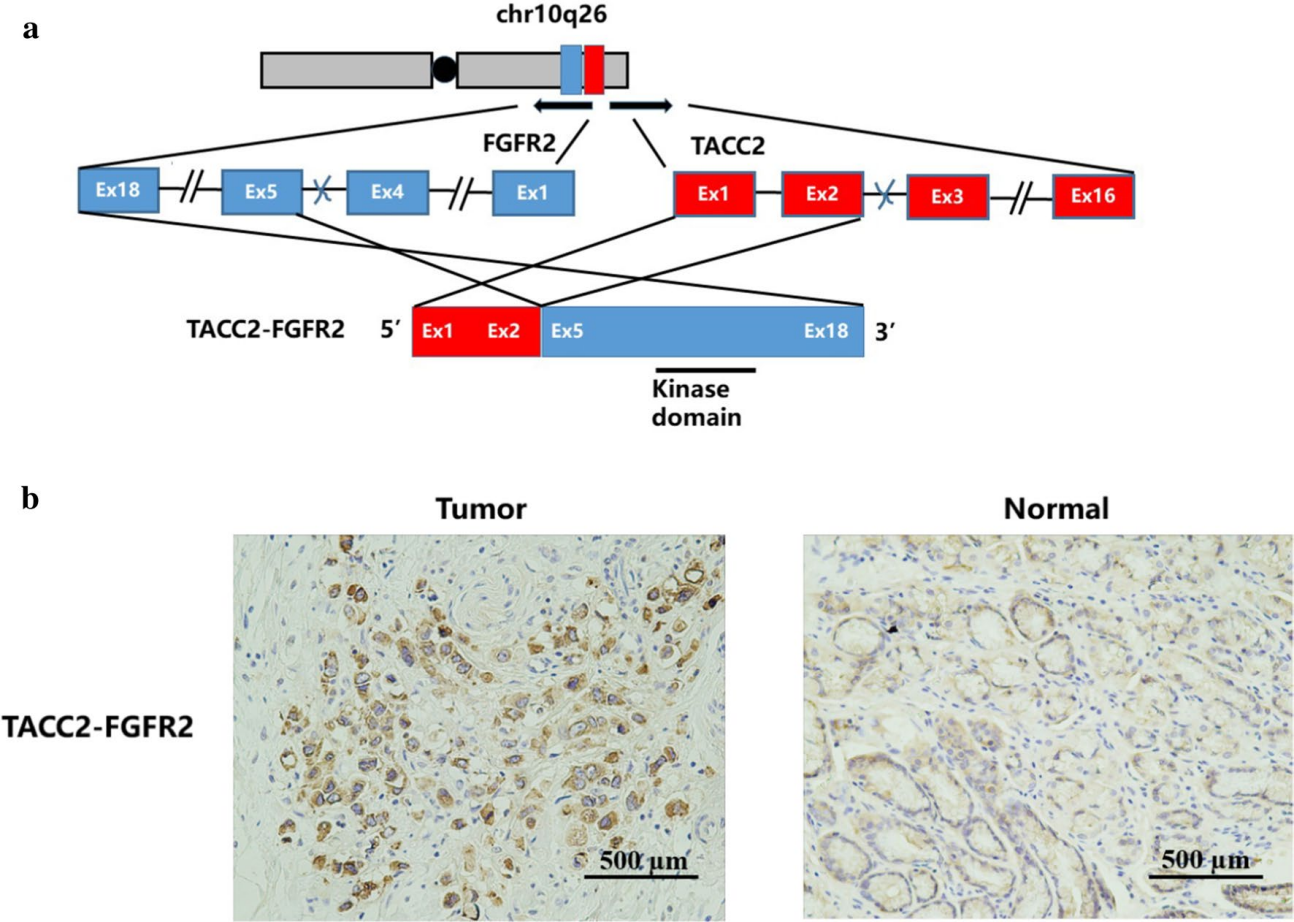
**a** Illustration of breakpoint of TACC2 and FGFR2. Exons (Ex) of TACC2 and FGFR2 are indicated with red and blue, respectively. **b** Representative images of FGFR2 staining for PC GC patients with TACC2-FGFR2 rearrangement with tumor area (left), adjacent normal surface epithelial area (right). Scale bar = 500 µm

Fluorescence in situ hybridization (FISH) and RNA sequencing (RNA-seq) was performed in two samples with FGFR2 rearrangements (FGFR2/VTI1A and FGFR2/TACC2). FGFR2/VTI1A and FGFR2/TACC2 rearrangements could both be detected by FISH in corresponding patient’s samples (Additional file 1: Figure S2). TACC2-FGFR2 fusion was verified in the form of TACC2 (exon1–2) -FGFR2 (exon5–18) by RNA-seq (Fig. 3a, Additional file 1: Figure S3). However, no RNA product of FGFR2-VTI1A rearrangement was detected by RNA-seq. We presumed that it might be due to no RNA product was transcribed by FGFR2-VTI1A rearrangement indeed. Next, we evaluated the expression level of FGFR2 protein in patient’s surgical samples with TACC2-FGFR2 using immunohistochemistry (IHC), finding increased FGFR2 expression in tumor area compared to adjacent normal surface epithelial area (Fig. 3b).

**Fig. 4:**
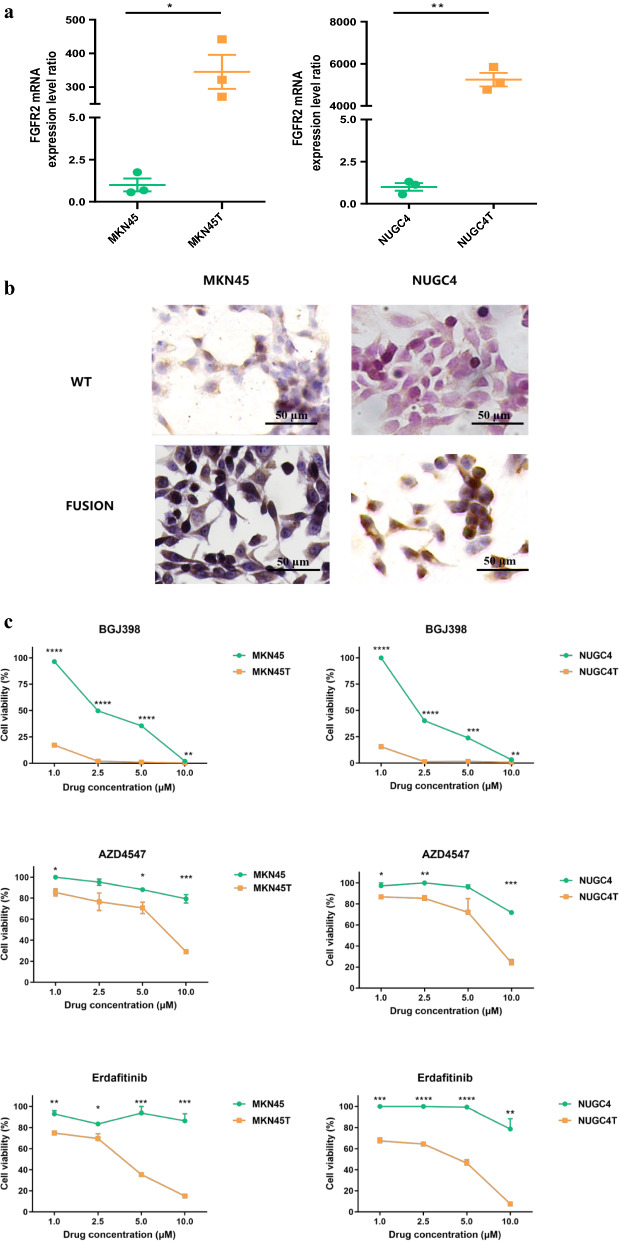
**a** qRT-PCR for the TACC2-FGFR2 fusion transcript in MKN45, NUGC4 cells and corresponding TACC2-FGFR2-expressing cells. Data are presented as mean ± SEM. * means* P* < 0.05, ** means *P* < 0.01. **b** IHC of MKN45, NUGC4 cells and corresponding TACC2-FGFR2-expressing cells for FGFR2 protein. Scale bar = 50 µm. MKN45T: TACC2-FGFR2-expression MKN45 cells. NUGC4T: TACC2-FGFR2-expression NUGC4 cells. **c** Sensitivity of MKN45, NUGC4 cells transfected with TACC2-FGFR2 or control plasmids to FGFR2 inhibitors, including BGJ398, AZD4547 and Erdafitinib. MKN45T: TACC2-FGFR2-expression MKN45 cells. NUGC4T: TACC2-FGFR2-expression NUGC4 cells

To further investigate TACC2-FGFR2 fusion in PC GC, we stably expressed TACC2-FGFR2 in gastric cancer cell lines MKN45 and NUGC4. Using qRT-PCR, we found FGFR2 mRNA levels were increased in TACC2-FGFR2-expressing MKN45 and NUGC4 cells (Fig. 4a). Moreover, IHC showed TACC2-FGFR2 upregulated FGFR2 protein expression in TACC2-FGFR2-expressing MKN45 and NUGC4 cells. (Fig. 4b).

To figure out the role of TACC2-FGFR2 fusion in targeted therapy for GC, we treated MKN45, NUGC4 cells and TACC2-FGFR2-expressing MKN45, NUGC4 cells with FGFR2 inhibitors, including BGJ398, AZD4547 and Erdafitinib. TACC2-FGFR2-expressing MKN45 and NUGC4 cells are more sensitive to all these three FGFR2 inhibitors (Fig. 4c).

## Discussion

GC is a phenotypically and molecularly highly heterogeneous disease. Intratumoral, intrapatient and interpatient heterogeneity in GC remains a crucial barrier for targeted therapies [2]. PC GC is a unique subtype of GC, tending to metastasis and with poor prognosis [13, 14]. None of current therapies showed satisfying results [4]. Therefore, there is a critical need to develop new efficacious therapeutic agents for PC GC. Molecular characteristics are becoming more and more important for GC treatment. Several molecular GC classification systems have been proposed these years for better clinical treatments [8, 9]. A few papers have focused on genomic alterations of PC GC and presented significantly mutated genes identified are TP53, APC, KIT, EGFR, PIK3CA, CTNNB1, ARID1A, and CDH1 [13, 15–18]. Our results showed the top 10 most frequent altered genes among PC GC were TP53 (48%), CDH1 (31%), ARID1A (14%), FGFR2 (14%), ERBB2 (9%), CDKN2A (9%), FGF3 (8%), LRP1B (9%), FGF19 (8%) and FGF4 (8%), consistent with previous research.

However, these molecular characteristics of PC GC patients are still unclear for treatment and prognosis. To better understand the molecular characteristics of PC GC, we respectively analyzed SNVs and INDELs, CNVs and gene fusions and rearrangements. We found that 20% (13/64) PC GC patients harboring somatic gene rearrangements. And, patients with rearrangements (n = 9) had a shorter overall survival (OS) in comparison with patients without any gene rearrangement (n = 19) (16.0 months vs 21.0 months, *P* = 0.043) (Fig. 3b). It may owe to gene rearrangements could form severe cancer driving structural variants (SV), including insertions, deletions, tandem duplications, inversions, translocations, and more complex rearrangements [19]. This is an indication that gene rearrangements play an important role in PC GC development and prognosis. Gene rearrangement might be an adverse prognostic factor for PC GC patients and more effective therapies targeting gene rearrangements should be studied for PC GC.

FGFR2, known as fibroblast growth factor receptor-2, is a transmembrane tyrosine kinase receptor, regulating cell proliferation, survival, migration and angiogenesis [20]. Genetic alterations in FGFR2, including gene amplification, mutations or rearrangements may dysregulate the FGF signaling pathway, influence the development and progression of various cancers by activating the downstream PI3K–AKT and MAPK–ERK pathways [21]. Previous study demonstrated that FGFR2 overexpression, mainly due to FGFR2 gene amplification, is associated with poor pathological features, including deeper tumor invasion, more LN metastasis, advanced tumor stage, and worse survival in GC [22]. And, many researches indicated that the incidence of FGFR2 overexpression might differ between histological subtypes according to Lauren’s classification [21]. Hattori found FGFR2 overexpression in 52.6% (20/38) diffuse-type GCs, but in none of 11 intestinal-type GCs [23]. PC GC tend to be diffuse-type according to Lauren’s classification, however, no study verified the situation of FGFR2 genomic alterations in PC GC. Our results firstly showed that FGFR2 is more frequently mutated in PC GC than non-PC GC (14% vs. 6%, P = 0.037), including 12.5% CNVs. Several small-molecule inhibitors and antibodies for FGFR2 are under clinical trials [24, 25]. Recently, Catenacci [25] verified bemarituzumab, an IgG1 antibody specific for the FGFR2b receptor, seemed to be well tolerated and demonstrated single-agent activity as lateline therapy in patients with advanced-stage gastric and gastroesophageal junction adenocarcinoma (GEA). Moreover, bemarituzumab is currently being evaluated in combination with chemotherapy in a phase III trial as front-line therapy for patients with high FGFR2b-overexpressing advanced-stage GEA. Preliminary results showed that bemarituzumab, added to mFOLFOX6 chemotherapy, led to clinically meaningful and statistically significant improvements in PFS (9.5 m vs. 7.4 m, *P* = 0.0727), OS (not reach vs. 12.9 m, *P* = 0.0268) and ORR (47% vs. 33%). And, the higher the FGFR2 expression, the better the prognosis [24]. FGFR2 inhibitors may be a good choice for PC GC with FGFR2 CNVs.

In addition, PC GC patients not only have more FGFR2 CNVs, but also have recurrent FGFR2 rearrangement (FGFR2/VTI1A and FGFR2/TACC2) in our study. FGFR rearrangements present in 13% to 17% of intrahepatic cholangiocarcinoma and represent driver mutations [26]. Several pre-clinical studies and clinical trials have demonstrated that FGFR2 rearrangements in cholangiocarcinoma can predict tumor sensitivity to FGFR2 inhibitors and become an important therapy in these highly selected patients [27–29]. To verify whether FGFR2 rearrangements can be a marker for targeted therapy in PC GC patients, we transfected TACC2-FGFR2 construct into GC cell lines (MKN45 and NUGC4), which has not been studied in PC GC before. We chose FGFR2 inhibitors, including AZD4547, Erdafitinib, and BGJ398 to treat GC cell lines with or without TACC2-FGFR2 fusion, and got similar results. Recently, many FGFR2 inhibitors are in the pipeline, these three drugs belonged to the first FGFR2 inhibitors studied. AZD4547 has been used in clinical trials of gastric adenocarcinoma [30], and Erdafitinib (Balversa™, Janssen Pharmaceutical Companies) [31] and BGJ398 (Infigratinib) [27, 32] was or will be approved by FDA for treatment of tumor with FGFR2 alterations. Our results firstly verified that the FGFR2 mRNA and protein expression level (Fig. 4b, c) were increased in GC cells with FGFR2 rearrangement and cells became more sensitive to FGFR2 inhibitors.

## Conclusion

In conclusion, we firstly identified FGFR2 alteration was more frequently among PC GC than non-PC GC, including CNVs and rearrangements. Moreover, we verified TACC2-FGFR2 fusion could increase FGFR2 expression in mRNA and protein level, and GC cell lines with TACC2-FGFR2 fusion were more sensitive to FGFR2 inhibitors. All these results suggested that FGFR2 may be a potential therapeutic target for PC GC.

## Supplementary Information

**Additional file 1: Figure S1.** Schema diagram of NGS process. *NGS* next generation sequencing. **Table S1.** Univariate and multivariate analyses for PC GC patients. **Figure S2.** FISH detection of the FGFR2 fusion. Yellow arrows indicate separate location of different FGFR2 exons (red and green). Scale bar = 5 µm. **Figure S3.** RNA-sequence reads map of TACC2-FGFR.

## Data Availability

The data described in this manuscript are contained in published articles or available from the corresponding author upon reasonable request.

## Abbreviations

AJCC: American Joint Committee on Cancer
CCK-8: Cell counting kit-8
CIN: Chromosomal instability
CNVs: Copy number variants
EBV: Epstein–Barr virus
FFPE: Formalin fixed paraffin embedded
FISH: Fluorescence in situ hybridization
GC: Gastric cancer
GEA: Gastroesophageal junction adenocarcinoma
GS: Genomically stable
IHC: Immunohistochemistry
Indels: Short and long insertions/deletions
MMR: Mismatch repair
MSI: Microsatellite instability
MSI-H: Microsatellite instability-high
NGS: Next-generation sequencing
OS: Overall survival
PC: Poorly cohesive
qRT-PCR: Quantitative real time polymerase chain reaction
RNA-seq: RNA sequencing
SNVs: Single nucleotide variants
SV: Structural variants
TCGA: The Cancer Genome Atlas
TMB: Tumor mutation burden
TMB-H: Tumor mutational burden-high
TMB-L: Tumor mutational burden-low
TP53: Tumor protein 53

## References

[1] Bray F, Ferlay J, Soerjomataram I, Siegel RL, Torre LA, Jemal A (2018). Global cancer statistics 2018: GLOBOCAN estimates of incidence and mortality worldwide for 36 cancers in 185 countries. CA Cancer J Clin.

[2] Smyth EC, Nilsson M, Grabsch HI, van Grieken NCT, Lordick F (2020). Gastric cancer. The Lancet.

[3] Mariette C, Carneiro F, Grabsch HI, van der Post RS, Allum W, de Manzoni G (2019). Consensus on the pathological definition and classification of poorly cohesive gastric carcinoma. Gastric Cancer.

[4] Wagner AD, Grabsch HI, Mauer M, Marreaud S, Caballero C, Thuss-Patience P (2019). EORTC-1203-GITCG - the "INNOVATION"-trial: Effect of chemotherapy alone versus chemotherapy plus trastuzumab, versus chemotherapy plus trastuzumab plus pertuzumab, in the perioperative treatment of HER2 positive, gastric and gastroesophageal junction adenocarcinoma on pathologic response rate: a randomized phase II-intergroup trial of the EORTC-Gastrointestinal Tract Cancer Group, Korean Cancer Study Group and Dutch Upper GI-Cancer group. BMC Cancer.

[5] Sarriugarte Lasarte A, GarcIa Alberdi E, Martinez Indart L, Gutierrez Grijalba O, Albarez Abad I, Guerra Lerma M (2020). "From Lauren's diffuse gastric cancer to WHO's poorly cohesive carcinoma." Clinicopathological and prognostic characteristics. Rev Esp Enferm Dig.

[6] Tan IB, Ivanova T, Lim KH, Ong CW, Deng N, Lee J, et al. Intrinsic subtypes of gastric cancer, based on gene expression pattern, predict survival and respond differently to chemotherapy. Gastroenterology. 2011;141:476–85, 85 e1–11. 10.1053/j.gastro.2011.04.042 PMC3152688 21684283

[7] Lei Z, Tan IB, Das K, Deng N, Zouridis H, Pattison S (2013). Identification of molecular subtypes of gastric cancer with different responses to PI3-kinase inhibitors and 5-fluorouracil. Gastroenterology.

[8] Cancer Genome Atlas Research N (2014). Comprehensive molecular characterization of gastric adenocarcinoma. Nature.

[9] Cristescu R, Lee J, Nebozhyn M, Kim KM, Ting JC, Wong SS (2015). Molecular analysis of gastric cancer identifies subtypes associated with distinct clinical outcomes. Nat Med.

[10] Alborelli I, Leonards K, Rothschild SI, Leuenberger LP, Savic Prince S, Mertz KD (2020). Tumor mutational burden assessed by targeted NGS predicts clinical benefit from immune checkpoint inhibitors in non-small cell lung cancer. J Pathol.

[11] Tappeiner E, Finotello F, Charoentong P, Mayer C, Rieder D, Trajanoski Z (2017). TIminer: NGS data mining pipeline for cancer immunology and immunotherapy. Bioinformatics.

[12] Rosenthal R, McGranahan N, Herrero J, Taylor BS, Swanton C (2016). DeconstructSigs: delineating mutational processes in single tumors distinguishes DNA repair deficiencies and patterns of carcinoma evolution. Genome Biol.

[13] Huang KH, Chen MH, Fang WL, Lin CH, Chao Y, Lo SS (2020). The clinicopathological characteristics and genetic alterations of signet-ring cell carcinoma in gastric cancer. Cancers (Basel).

[14] Lee D, Ham IH, Son SY, Han SU, Kim YB, Hur H (2017). Intratumor stromal proportion predicts aggressive phenotype of gastric signet ring cell carcinomas. Gastric Cancer.

[15] Shu Y, Zhang W, Hou Q, Zhao L, Zhang S, Zhou J (2018). Prognostic significance of frequent CLDN18-ARHGAP26/6 fusion in gastric signet-ring cell cancer. Nat Commun.

[16] Xu B, Liu F, Liu Q, Shi T, Wang Z, Wu N (2020). Highly expressed Claudin18.2 as a potential therapeutic target in advanced gastric signet-ring cell carcinoma (SRCC). J Gastrointest Oncol.

[17] Wang K, Yuen ST, Xu J, Lee SP, Yan HH, Shi ST (2014). Whole-genome sequencing and comprehensive molecular profiling identify new driver mutations in gastric cancer. Nat Genet.

[18] Kwon CH, Kim YK, Lee S, Kim A, Park HJ, Choi Y (2018). Gastric poorly cohesive carcinoma: a correlative study of mutational signatures and prognostic significance based on histopathological subtypes. Histopathology.

[19] Yang L, Luquette LJ, Gehlenborg N, Xi R, Haseley PS, Hsieh CH (2013). Diverse mechanisms of somatic structural variations in human cancer genomes. Cell.

[20] Turner N, Grose R (2010). Fibroblast growth factor signalling: from development to cancer. Nat Rev Cancer.

[21] Ahn S, Lee J, Hong M, Kim ST, Park SH, Choi MG (2016). FGFR2 in gastric cancer: protein overexpression predicts gene amplification and high H-index predicts poor survival. Mod Pathol.

[22] Kim HS, Kim JH, Jang HJ, Han B, Zang DY (2019). Pathological and prognostic impacts of FGFR2 overexpression in gastric cancer: a meta-analysis. J Cancer.

[23] Hattori Y, Itoh H, Uchino S, Hosokawa K, Ochiai A, Ino Y (1996). Immunohistochemical detection of K-sam protein in stomach cancer. Clin Cancer Res.

[24] Wainberg ZA, Enzinger PC, Kang Y-K, Yamaguchi K, Qin S, Lee K-W, Oh SC, Li J, Turk HM. A double-blind randomized study of bemarituzumab (bema) plus mFOLFOX6 versus placebo plus mFOLFOX6 as first-line treatment for advanced gastric/gastroesophageal junction cancer (FIGHT). In: ASCO gastrointestinal cancer symposium. 2021;LBA160.

[25] Catenacci DVT, Rasco D, Lee J, Rha SY, Lee K-W, Bang YJ, Bendell J, Enzinger P, Marina N, Xiang H, Deng W, Powers J, Wainberg ZA (2020). Phase I escalation and expansion study of bemarituzumab (FPA144) in patients with advanced solid tumors and FGFR2b-selected gastroesophageal adenocarcinoma. J Clin Oncol.

[26] Graham RP, Barr Fritcher EG, Pestova E, Schulz J, Sitailo LA, Vasmatzis G (2014). Fibroblast growth factor receptor 2 translocations in intrahepatic cholangiocarcinoma. Hum Pathol.

[27] Javle M, Lowery M, Shroff RT, Weiss KH, Springfeld C, Borad MJ (2018). Phase II study of BGJ398 in patients with FGFR-altered advanced cholangiocarcinoma. J Clin Oncol.

[28] Ross JS, Wang K, Gay L, Al-Rohil R, Rand JV, Jones DM (2014). New routes to targeted therapy of intrahepatic cholangiocarcinomas revealed by next-generation sequencing. Oncologist.

[29] Farshidfar F, Zheng S, Gingras MC, Newton Y, Shih J, Robertson AG (2017). Integrative genomic analysis of cholangiocarcinoma identifies distinct IDH-mutant molecular profiles. Cell Rep.

[30] Van Cutsem E, Bang YJ, Mansoor W, Petty RD, Chao Y, Cunningham D (2017). A randomized, open-label study of the efficacy and safety of AZD4547 monotherapy versus paclitaxel for the treatment of advanced gastric adenocarcinoma with FGFR2 polysomy or gene amplification. Ann Oncol.

[31] Loriot Y, Necchi A, Park SH, Garcia-Donas J, Huddart R, Burgess E (2019). Erdafitinib in locally advanced or metastatic urothelial carcinoma. N Engl J Med.

[32] Nogova L, Sequist LV, Perez Garcia JM, Andre F, Delord JP, Hidalgo M (2017). Evaluation of BGJ398, a fibroblast growth factor receptor 1–3 kinase inhibitor, in patients with advanced solid tumors harboring genetic alterations in fibroblast growth factor receptors: results of a global phase I, dose-escalation and dose-expansion study. J Clin Oncol.

