# Supplementary material for: FGFR2 alteration as a potential therapeutic target in poorly cohesive gastric carcinoma
Source: J Transl Med. 2021 Sep 22;19:401. doi: 10.1186/s12967-021-03079-8 (PMC8459493; doi:10.1186/s12967-021-03079-8)
Supplement: Supplementary file 1 — Additional file 1: Figure S1. Schema diagram of NGS process. NGS next generation sequencing. Table S1. Univariate and multivariate analyses for PC GC patients. Figure S2. FISH detection of the FGFR2 fusion. Yellow arrows indicate separate location of different FGFR2 exons (red and green). Scale bar = 5 µm. Figure S3. RNA-sequence reads map of TACC2-FGFR. [file 12967_2021_3079_MOESM1_ESM.docx]

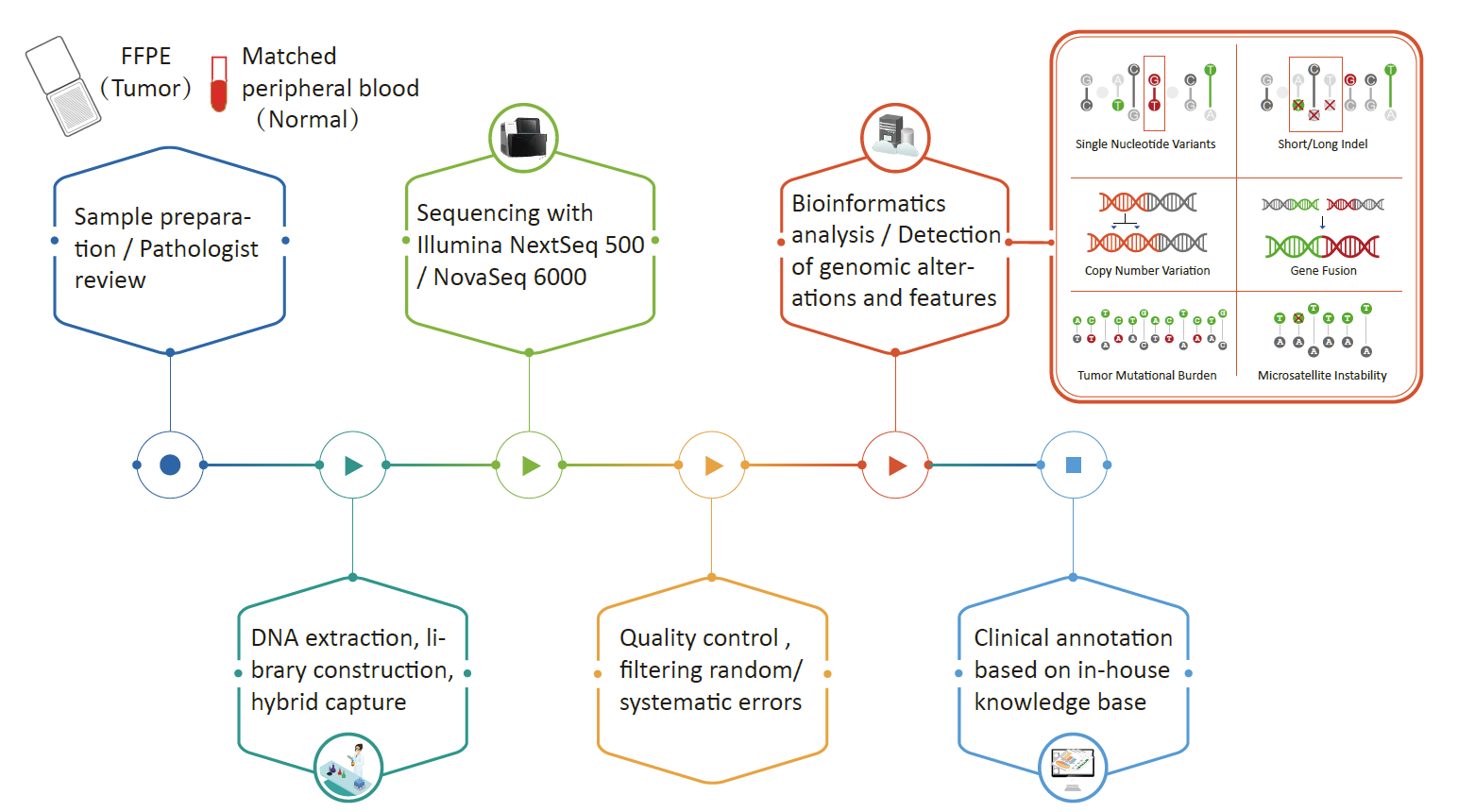


**Figure S1:** Schema diagram of NGS process. *NGS* next generation sequencing.

| **Variable** | **Univariate Cox Analysis** | | |  | **Multivariate Cox Analysis** | | |
| --- | --- | --- | --- | --- | --- | --- | --- |
|  | **HR** | **95% CI** | ***P* value** |  | **HR** | **95% CI** | ***P* value** |
| **Rearrangement** |  |  |  |  |  |  |  |
| Without | Ref |  |  |  | Ref |  |  |
| With | 2.384 | 0.993-5.723 | 0.052 |  | 0.955 | 0.246-3.712 | 0.947 |
| **Age** |  |  |  |  |  |  |  |
| <60 years | Ref |  |  |  | Ref |  |  |
| ≥60 years | 2.630 | 1.104-6.268 | *0.029* |  | 3.083 | 1.114-8.531 | *0.030* |
| **Gender** |  |  |  |  |  |  |  |
| Male | Ref |  |  |  |  |  |  |
| Female | 0.591 | 0.246-1.421 | 0.240 |  |  |  |  |
| **AJCC** |  |  |  |  |  |  |  |
| IIIA-B | Ref |  |  |  | Ref |  |  |
| IIIC | 2.506 | 1.026-6.122 | *0.044* |  | 0.692 | 0.084-5.680 | 0.732 |
| **T stage** |  |  |  |  |  |  |  |
| 3 | Ref |  |  |  |  |  |  |
| 4 | 1.418 | 0.619-3.249 | 0.409 |  |  |  |  |
| **N stage** |  |  |  |  |  |  |  |
| 1-3a | Ref |  |  |  | Ref |  |  |
| 3b | 3.789 | 1.507-9.527 | *0.005* |  | 9.068 | 0.492-167.029 | 0.138 |
| **Tumor size** |  |  |  |  |  |  |  |
| ≤4cm | Ref |  |  |  |  |  |  |
| >4cm | 1.533 | 0.633-3.712 | 0.344 |  |  |  |  |
| **Tumor location** |  |  |  |  |  |  |  |
| Upper | Ref |  |  |  | Ref |  |  |
| Middle | 1.106 | 0.380-3.225 | 0.853 |  | 3.369 | 0.798-14.218 | 0.098 |
| Lower | 0.599 | 0.200-1.797 | 0.361 |  | 1.531 | 0.424-5.532 | 0.516 |
| Overlap | 4.543 | 1.089-18.948 | *0.038* |  | 2.197 | 0.454-10.631 | 0.328 |
| **PD-L1** |  |  |  |  |  |  |  |
| Negative | Ref |  |  |  |  |  |  |
| Positive | 0.985 | 0.441-2.198 | 0.970 |  |  |  |  |
| **PD-1** |  |  |  |  |  |  |  |
| Negative | Ref |  |  |  |  |  |  |
| Positive | 0.509 | 0.172-1.504 | 0.222 |  |  |  |  |
| **CD3 number** |  |  |  |  |  |  |  |
| Low | Ref |  |  |  |  |  |  |
| High | 0.836 | 0.374-1.867 | 0.661 |  |  |  |  |

*AJCC* American Joint Committee on Cancer, *GC* gastric cancer, *PC* poorly cohesive

**Table S1:** Univariate and multivariate analyses for PC GC patients


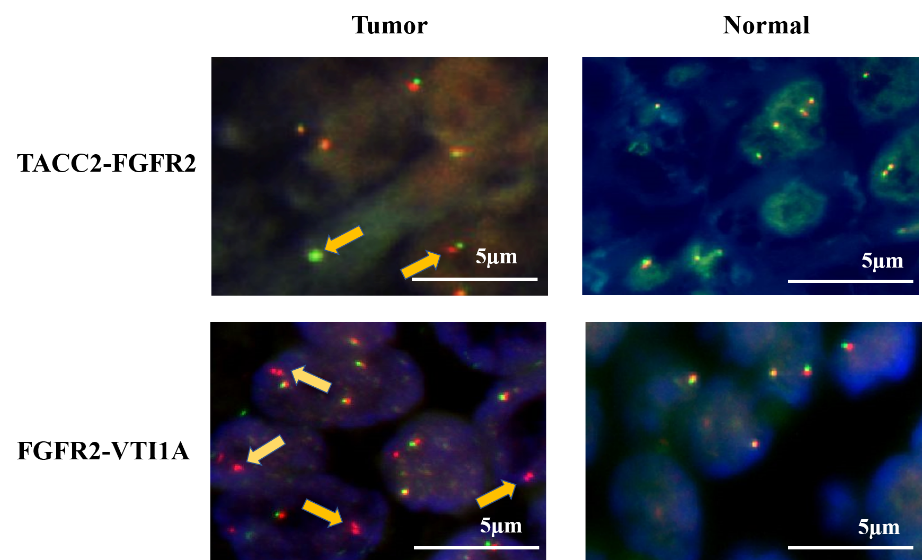


**Figure S2:** FISH detection of the FGFR2 fusion. Yellow arrows indicate separate location of different FGFR2 exons (red and green). Scale bar = 5 µm.


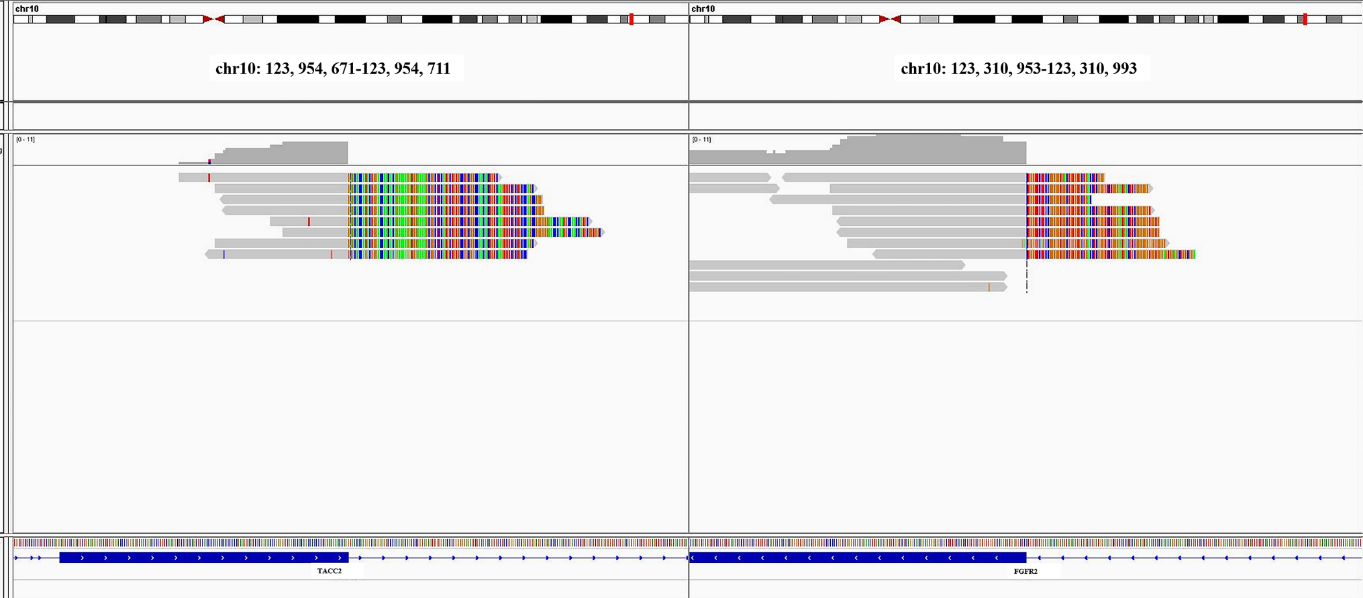


**Figure S3:** RNA-sequence reads map of TACC2-FGFR.
